# Developmental Pharmacokinetics and Safety of Ibuprofen and Its Enantiomers in the Conventional Pig as Potential Pediatric Animal Model

**DOI:** 10.3389/fphar.2019.00505

**Published:** 2019-05-09

**Authors:** Joske Millecam, Thomas van Bergen, Stijn Schauvliege, Gunther Antonissen, Ann Martens, Koen Chiers, Ronette Gehring, Elke Gasthuys, Johan Vande Walle, Siska Croubels, Mathias Devreese

**Affiliations:** ^1^Laboratory of Pharmacology and Toxicology, Department of Pharmacology, Toxicology and Biochemistry, Faculty of Veterinary Medicine, Ghent University, Ghent, Belgium; ^2^Department of Surgery and Anesthesiology of Domestic Animals, Faculty of Veterinary Medicine, Ghent University, Ghent, Belgium; ^3^Department of Pathology, Bacteriology and Avian Diseases, Faculty of Veterinary Medicine, Ghent University, Ghent, Belgium; ^4^Institute for Risk Assessment, Faculty of Veterinary Medicine, Utrecht University, Utrecht, Netherlands; ^5^Department of Internal Medicine and Pediatrics, Faculty of Medicine and Health Sciences, Ghent University, Ghent, Belgium

**Keywords:** ibuprofen, pig, juvenile, enantiomers, pharmacokinetics, animal model

## Abstract

Pediatric drug development, especially in disease areas that only affect children, can be stimulated by using juvenile animal models not only for general safety studies, but also to gain knowledge on the pharmacokinetic and pharmacodynamic properties of the drug. Recently, the conventional growing piglet has been suggested as juvenile animal model. However, more studies with different classes of drugs are warranted to make a thorough evaluation whether the juvenile pig might be a suitable preclinical animal model. Ibuprofen is one of the most widely used non-steroidal anti-inflammatory drugs in human. The present study determined the PK parameters, gastro-intestinal and renal safety of 5 mg/kg BW ibuprofen after single intravenous, single oral and multiple oral administration to each time eight pigs (four males, four females) aging 1, 4, 8 weeks and 6–7 months. Oral administration was performed via a gastrostomy button. A jugular catheter was used for intravenous administration and blood sampling. To assess NSAID induced renal toxicity, renal function was evaluated using iohexol and *p*-aminohippuric acid as markers for glomerular filtration rate and renal plasma flow, respectively. After the trial, necropsy and histology was performed to evaluate macroscopic and microscopic gastro-intestinal as well as renal lesions. Both enantiomers, R-ibuprofen and S-ibuprofen, were determined in plasma using an in-house developed and validated UHPLC-MS/MS method. Pharmacokinetic parameters were estimated using compartmental analysis. Clearance and volume of distribution of total ibuprofen and both enantiomers increased with age as was observed in human. The rate of stereochemical conversion decreased with age. Multiple oral dosing decreased the absolute oral bioavailability and maximum plasma concentration of R-ibuprofen and food consumption did not influence drug absorption. Based on the limited available pediatric literature, the current study might suggest the conventional pig as suitable animal model to evaluate NSAIDs for pediatric use.

## Introduction

Since the implementation of the Pediatric Investigation Plan (PIP) and the Pediatric Safety Plan (PSP) by, respectively, the European Medicines Agency (EMA) and the Food and Drug Administration (FDA), the number of clinical trials in children increased, leading to more and better availability of medicines for children. However, since the PIP and PSP are driven from the adult drug development path, little progress has been made in diseases that only affect children or where the disease shows biological differences between adults and children (Califf, 2016; EMA, 2017). The use of juvenile animal models might bridge that gap. Despite the increase in juvenile animal trials thanks to the pediatric legislations, almost all juvenile studies mentioned in the PIPs from 2007 to mid-2017 were general toxicology studies (Baldrick, 2018). Besides toxicology studies, there is a need for more pharmacokinetic (PK) and pharmacodynamic (PD) juvenile studies in the desired age category without any previous adult human or animal data. This would stimulate pediatric drug development in diseases that only affect children, or have a different pathogenesis compared to adults. Selecting the most appropriate animal species is crucial and the rat (57.5%) is still the most commonly used juvenile species, followed by dog (8%), mouse (4.5%), monkey (4%), pig (2%), sheep (1%), rabbit (1%), and hamster (0.5%). Unfortunately, in 21.5% of the cases, no species was mentioned (Baldrick, 2018). However, this does not mean that the rat is the most appropriate animal model to evaluate pediatric PK/PD and safety characteristics. The rat is often preferred due to the availability of a large historical dataset (De Schaepdrijver et al., 2013). Nevertheless, selection of the animal species should be based on anatomical and physiological developmental similarities and differences between the juvenile animal and the pediatric population of interest, technical requirements, and the properties of the drug (De Schaepdrijver et al., 2013).

Although the conventional pig is not yet readily used in preclinical research, PK/PD and safety studies for adults have already been performed successfully (Swindle et al., 2012; Helke and Swindle, 2013; Yoshimatsu et al., 2016). Pigs do display a high level of anatomical and physiological similarities with human regarding the organs involved in absorption, distribution, metabolism and excretion (ADME) of drugs. Moreover, growing piglets display similar maturational processes as seen in children (Gasthuys et al., 2016, 2017a; Millecam et al., 2018). Recently, Gasthuys et al. (2018) performed a PK/PD study of desmopressin in growing piglets and found the piglet to be an appropriate animal model to predict the clearance of desmopressin in humans. Nevertheless, other drug classes need to be evaluated to verify whether the growing piglet might be a good model for the pediatric population in general.

It has been over 50 years since the discovery of the non-steroidal anti-inflammatory drug (NSAID) ibuprofen (IBU). Due to its relatively low risks for gastro-intestinal, hepato-renal, and other adverse events at over-the-counter doses (<1200 mg/day), a short elimination half-live compared to other NSAIDs and thus little propensity to accumulate systemically, IBU is one of the most used drugs for treatment of acute pain and fever in humans (Rainsford, 2011). In children, the use of IBU is approved from 3 months old onwards and is widely used for the same indications as in adults, namely treatment of inflammation, mild-to-moderate pain and fever. Safety and efficacy studies are widely available in the pediatric population, but specific PK studies are limited, especially regarding the enantiomers of IBU. On top of that, most studies report general mean values of PK parameters for the study population as a whole with a broad age range of included children or they have arbitrary age groups making it difficult to correctly interpret the data. Moreover, a detailed and reliable plasma concentration/therapeutic effect relationship for IBU in the pediatric population is currently lacking (de Martino et al., 2017). A thorough understanding of the developmental PK parameters could provide more information regarding the occurrence of adverse events and lead to better prediction of efficacy. Especially since S-ibuprofen (S-IBU) is the pharmacologically active enantiomer and each enantiomer can lead to different adverse events. In general, the limited PK data available do suggest similar PK properties between children younger than 12 years of age and young-to-middle aged adults (Rainsford, 2009). Rey et al. (1994) found the plasma concentrations of S-IBU to be lower than those of R-ibuprofen (R-IBU) in infants (6–18 months of age), indicating impaired conversion from R- to S-IBU and/or higher clearance of S-IBU. The clearance rate is believed to be higher in children up to about 5 years of age and declines in older age groups (Rainsford, 2009). As it is from an ethical point of view difficult to perform full PK studies in growing children, the conventional piglet might be a good substitute to gain this developmental PK data.

The aim of the current study was to determine the PK parameters of IBU after single intravenous, single oral and multiple oral administrations at therapeutic doses in conventional pigs of different ages. Next, gastro-intestinal and renal toxicity was evaluated to assess whether IBU could be safely administered to pigs as a model for children. All trials were performed in pigs aging 1, 4, 8 weeks and 6–7 months. These age groups correspond with the main age categories of the human pediatric population, namely neonate, infant, child, and adolescent (Gad, 2015; Gasthuys et al., 2016).

## Materials and Methods

### Animals

The current study was approved by the ethical committee of the Faculties of Veterinary Medicine and Bioscience Engineering of Ghent University (EC2016/105). Care and use of the animals were in full compliance with the national and European legislation on animal welfare and ethics (Eur-Lex, 2010; Flemish Government, 2017). Four age categories were included in the study corresponding with the main age groups of the human pediatric population, namely neonate, infant, child, and adolescent (Gad, 2015; Gasthuys et al., 2016). One-week-old piglets (3.0 ± 0.4 kg BW; Landrace × Large White × Maximus, RA-SE Genetics, Merkem, Belgium), 4-week-old piglets (7.0 ± 0.8 kg BW; Maximus, RA-SE Genetics, Bassilly, Belgium), 8-week-old piglets (20.1 ± 3.4 kg BW; Landrace × Large White, RA-SE Genetics and Convis, Ettelbruck, Luxembourg) and 6–7-months-old pigs (134 ± 4.6 kg BW for males and 142 ± 9.8 kg for females; Landrace × Large White, RA-SE Genetics and Convis, Ettelbruck, Luxembourg) were used representing the latter human age groups, respectively. Each age category consisted of 12 pigs (6 ♂/6 ♀), of which 8 pigs (4 ♂/4 ♀) received ibuprofen and 4 pigs (2 ♂/2 ♀) served as control. The pigs were randomly allocated to a treatment group taking an equal distribution of sex in all groups into account. All male pigs were intact. Since male and female pigs reach puberty at different ages and the influence of sex hormones on the PK of ibuprofen was of interest, the six male pigs were 6 months old, while the six female pigs were 7 months old (Van den Broeke et al., 2015). All pigs arrived at least 24 h prior to surgery at the test facility and were group-housed before surgical procedures in rescue decks (0.90 m × 1.40 m, Provimi, Rotterdam, Netherlands) (1-week-old), standard pig stables with partially slatted floors (2.30 m × 2.40 m) (3.5 and 8 weeks) or sow stables (0.65 m × 2.20 m) (6–7 months-old). A double lumen catheter was placed in the jugular vein and a gastrostomy button was inserted to facilitate blood sampling and multiple oral dosing, respectively according to Gasthuys et al. (2017b) and Millecam et al. (unpublished). After surgery, the animals were housed individually to avoid pen mates biting the catheters. All age groups had *ad libitum* access to feed (1 week: RescueMilk^®^, Provimi; 3.5 and 8 weeks: Biggistart Opti^®^, Aveve, Leuven, Belgium; 6–7 months: Optivo Pro^®^, Aveve) and water. Natural light was provided by translucent windows and the stable temperature was 24.3 ± 2.1°C during the whole conduct of the trials. Higher temperatures (30–35°C) in the rescue decks were obtained by heating lamps. One day prior to surgery, a cotton towel was given to the piglets (youngest three age categories) which was then passed on after surgery to mimic the smell of the other piglets when they were singly housed. The 1-week-old pigs could also hear, smell and see (Plexiglass^®^) each other. All stables were enriched with suspension chains, rubber toys, and balls which were rotated on a daily basis.

Prior and after surgery, all pigs were weighed on a daily basis for the whole conduct of the trial (10 days), except the 6–7 months old pigs who were only weighed the day of surgery. The pigs were intensively socialized to facilitate the handling with the catheter and button. Both lumens of the jugular catheter were flushed at least once daily with a sterile diluted heparin solution (1-week-old piglets: 0.04% v/v; 4- and 8-week-old piglets: 1% v/v; 6–7-months-old pigs: 2% v/v). Sealing caps and bandages were changed when needed and wound healing was monitored. The stomach button was flushed daily with tap water and the skin surrounding the stoma was visually inspected on a daily basis. The water and feed intake, body temperature and interaction with animal caretakers were monitored twice daily. Temperature was measured via a LifeChip^®^ (Allflex Europe SA, Vitré, France) placed in the left semitendinosus muscle during anesthesia. To evaluate possible early signs of inflammation, total white blood cell (WBC) count was performed daily, from the day after surgery till the end of the trial by taking 1 mL blood via the double lumen catheter in an K_3_EDTA collection tube (Vacutest^®^ Kima, Arzergrande, Italy). White blood cell count was performed by Medvet BVBA (Antwerp, Belgium). If the piglets showed more apathy and had a body temperature ≥ 40°C, they were treated with an intramuscular injection of 0.4 mg/kg BW of meloxicam (Metacam^®^ 5 mg/mL, Boehringer Ingelheim Vetmedica GmbH, Ingelheim am Rhein, Germany).

### Experimental Design of the Ibuprofen PK Study

**FIGURE 1:**
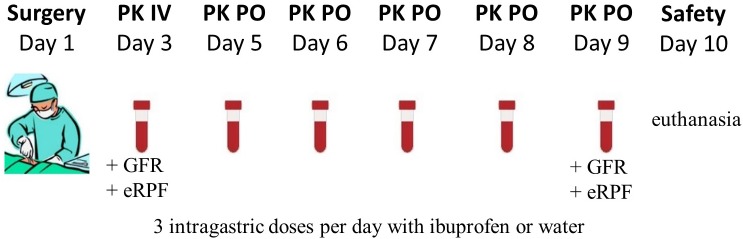
Graphical overview of the experimental setup. In total, 12 animals (PK: 4 ♂, 4 ♀; control: 2 ♂, 2 ♀) per age category (1, 4, 8 weeks and 6–7 months old) underwent surgery. The single intravenous dose (IV) ibuprofen was 5 mg/kg BW and the oral dose (PO) was 5 mg/kg three times a day. On day three and day 9, the glomerular filtration rate (GFR) and estimated renal plasma flow (eRPF) were determined.

The experimental design was identical for all four age categories and is graphically shown in Figure 1. The control pigs did not receive any IBU during the trial, but were sham-treated with water or NaCl solution for the oral and IV administration, respectively. After surgery, the pigs had 1 day to recover before the single dose intravenous PK study of 5 mg/kg BW IBU (Ibuprofenum, 50/50 ratio R/S-IBU, Fagron, Inc., Meer, Belgium) was initiated. The drug was dissolved in 0.9% NaCl (stock solution of 100 mg/mL) and administered IV using the proximal lumen of the jugular catheter. Next, one wash-out day was respected before starting the multiple dosing study with a pediatric IBU suspension (5 mg/kg BW; Ibuprofen EG^®^ 40 mg/mL, 50/50 ratio R/S-IBU, Eurogenerics, Brussels, Belgium). All pigs were fasted overnight before the first oral IBU administration, except for the 1-week-old piglets. The youngest age group was deprived of milk only 1 h before administration due to the risk of hypoglycemia. All pigs had again access to feed one and a half hour after administration (p.a.). Ibuprofen was given three times a day for five consecutive days. The dose interval was 6 h between the morning and the noon dose and between the noon and the evening dose. After each oral dose, the gastric tube was flushed with tap water (≥5 mL) to make sure all IBU entered the stomach. The control pigs received the same amount of tap water each time. Venous blood samples for PK analysis were taken on different time points through the distal lumen of the jugular catheter. The day of IV administration, blood was taken prior to and 5, 10, 20, 30, 45, 60 min and 1.5, 2, 2.5, 3, 4, 6, 8, and 24 h p.a. For oral multiple dose PK analysis, blood samples were drawn each time right before and 30 min p.a., except for the first (single dose fasted) and 13th (not fasted) oral dose where a full PK profile was obtained by more frequent sampling (0, 5, 10, 20, 30, 45, 60 min and 1.25, 1.5, 1.75, 2, 2.5, 3, 4, and 6 h p.a.). All blood samples were transferred into 4 mL K_3_EDTA collection tubes, immediately kept on ice and centrifuged for 10 min at 2095 *g*. Plasma was aliquoted, frozen and stored at < −15°C until analysis. Analytical determination of both IBU enantiomers was performed by an in-house developed and validated UHPLC-PDA method which is described in the Supplementary Material. On day 10, all pigs were euthanized by an IV injection of an overdose of pentobarbital (Sodium pentobarbital 20%^®^, Kela, Hoogstraten, Belgium). When the double-lumen catheter was no longer functional, euthanasia was performed by intramuscular injection with a mixture (1:1, 0.22 mL/kg) of xylazine hydrochloride (Xyl-M 2%^®^, VMD, Arendonk, Belgium) and tiletamine-zolazepam (Zoletil 100^®^, Virbac, Netherlands) followed by intracardiac injection of an overdose of pentobarbital.

### Evaluation of Gastro-Intestinal and Renal Toxicity

Possible physiological changes in kidney function were evaluated by measuring the glomerular filtration rate (GFR) and estimated renal plasma flow (eRPF) on the first day of IBU administration IV and the last day of the oral administration (Figure 1). GFR was measured through a single dose of 64.7 mg/kg BW of iohexol (0.1 mL/kg BW, Omnipaque^®^ 300 mg I/mL, GE Healthcare, Belgium). Estimated RPF was determined via a single dose of 10 mg/kg BW of *p*-aminohippuric acid (PAH, stock solution of 200 mg/mL in 0.9% NaCl solution, Sigma-Aldrich, Overijse, Belgium). Sampling points overlapped with those of IBU and were taken right before administration and 5, 10, 30, 60 min and 2, 3, 6, and 8 h after administration. Determination of iohexol and PAH in porcine plasma was performed using a validated UHPLC-MS/MS method (Dhondt et al., 2019). A more detailed description of this analytical method is given in the Supplementary Material.

During necropsy, macroscopic lesions were evaluated in the stomach and kidneys. The stomach was removed and opened along the greater curvature. After discarding stomach contents and rinsing the mucosa with water, possible macroscopic gastric lesions were scored according to the Lanza score (Table 1) (Lanza et al., 1985). Small samples of duodenum, jejunum, ileum, pars oesophagea, antrum, fundus, left and right kidney were fixed in 4% formaldehyde, embedded in paraffin, sectioned at 5 μm and stained with hematoxylin and eosin (HE) according to standard techniques. The grading scale for histological examination of the gastro-intestinal samples is given in Table 1 and adapted from Geboes et al. (2000). The samples were blinded before scoring. Renal samples were microscopically evaluated for papillary necrosis.

**Table 1:** Macroscopic and microscopic grading scale for the gastro-intestinal samples (Lanza et al., 1985; Geboes et al., 2000).

Macroscopic grade	
0	Intact mucosa
1	Redness and hyperemia in the mucosa
2	One or two erosions or hemorrhaging lesions
3	3–10 erosions or hemorrhaging lesions
4	>10 erosions or hemorrhaging lesions
**Microscopic grade**	
Grade 1	Lymphoid follicles in mucosae and submucosae
*Subgrade 1.0*	*No increase in lymphoid aggregates or follicles*
*Subgrade 1.1*	*Moderate increase in lymphoid aggregates (<5)*
*Subgrade 1.2*	*Severe increase in lymphoid aggregates (≥5) and/or 1 lymphoid follicle*
*Subgrade 1.3*	*≥2 lymphoid follicles*
Grade 2	Chronic inflammatory infiltrate in lamina propria
*Subgrade 2.0*	*No infiltration*
*Subgrade 2.1*	*Mild infiltration*
*Subgrade 2.2*	*Moderate infiltration*
*Subgrade 2.3*	*Severe infiltration*
Grade 3	Erosion or ulceration
*Subgrade 3.0*	*No erosion, ulceration, or granulation tissue*
*Subgrade 3.1*	*Recovering epithelium + adjacent inflammation*
*Subgrade 3.2*	*Probable erosion – focally stripped*
*Subgrade 3.3*	*Unequivocal erosion*
*Subgrade 3.4*	*Ulcer or granulation tissue*

### Pharmacokinetic Analysis

**FIGURE 2:**
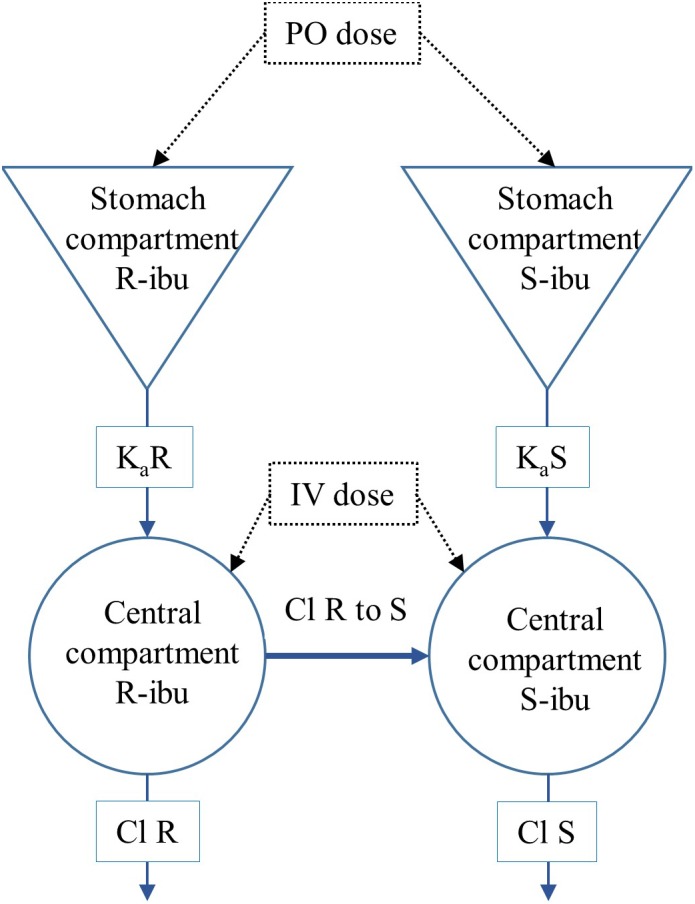
Representation of the 1-compartmental model for R- and S-ibuprofen (R-IBU and S-IBU). Cl R to S represents the systemic conversion of R-ibuprofen to S-ibuprofen.

All PK analyses were performed in Phoenix version 8.1 (Certara, Princeton, NJ, United States). Values below the LOQ of 0.25 μg/mL were excluded from the analysis. A 1-compartmental model was built taking the systemic conversion of R- to S-ibuprofen (R-to-S-IBU) into account (Figure 2). The dose of the individual enantiomers was considered half of the racemic dose of 5 mg/kg BW. Chiral inversion (Cl R to S) was estimated after IV administration and this value was fixed to estimate values of the PK parameters after oral administration. The plasma concentrations of total IBU were calculated from the sum of R- and S-IBU. The following primary and secondary PK parameters were calculated for IV and PO administration for total IBU, R- and S-IBU: clearance (IV only, Cl), apparent volume of distribution (IV only, V_d_), area under the plasma concentration time curve from time 0 and extrapolated to infinity (AUC_0→∞_), maximum plasma concentration for PO (C_max_), plasma concentration at time 0 for IV (C_0_), time to maximum plasma concentration (T_max_), elimination half-life (T_1/2_) and absorption rate constant (k_a_).

The absolute oral bioavailability (F) was estimated for every individual pig from the ratio of the areas under the plasma concentration time curve from time 0 to 3 h (AUC_0→3 h_) after PO and IV administration, calculated by non-compartmental analysis (NCA). The linear up log down trapezoidal method was used for AUC calculations.

The values of the PK parameters of iohexol and PAH were estimated using a two- and one-compartmental model, respectively. The Cl estimated from these models were defined as GFR and eRPF, respectively.

Allometric relationships were visually evaluated between Cl, V_d_, BW, GFR, and eRPF.

The accumulation ratio after multiple dosing was calculated using the AUC_0→6 h_ from the first and 13th oral dose according to Eq. 1.

$$\mathrm{accumulation ratio}=\frac{{\mathrm{AUC}}_{0\to 6h,\;\mathrm{dose}\;13}}{{\mathrm{AUC}}_{0\to 6h,\mathrm{dose}\;1}}(1).$$

### Statistical Analysis

All s MA, United States). In order to evaluate the effect of age and gender on the values of different PK parameters, a one-way nested ANOVA was performed (*p* < 0.05). Normality of the data was checked using Levene’s test. If the data did not met the criteria of normality (*p* < 0.01), a log transformation was performed. *Post hoc* analysis was done using Tukey’s HSD (Honestly Significant Difference) test. Evaluation of the same PK parameter between IV and the first PO administration and between the first and fifth day of PO administration was done using a pairwise *t*-test (*p* < 0.05). The significant differences (*p* < 0.05) between the same PK parameters for R- and S-IBU were evaluated using a Student’s *t*-test for every age group individually.

To evaluate differences in age and treatment group regarding gastro-intestinal and kidney lesions, a Kruskal–Wallis test was performed on the sum of the macroscopic and microscopic scoring per tissue. If the Kruskal–Wallis test was significant (*p* < 0.05), a Dunn test (*p* < 0.025) was performed as *post hoc* test. Since two comparisons, namely age and treatment group, were made in the Dunn test, the significance level of 0.05 was divided by two, resulting in an alpha of 0.025. GFR and eRPF were compared between start and end of the trial for every age group using a pairwise *t*-test (*p* < 0.05). Finally, changes in body temperature and total amount of WBCs were evaluated using an univariate type III repeated-measures ANOVA. If Mauchly’s sphericity test was significant (*p* < 0.05) the Greenhouse–Geisser correction was applied.

## Results

### UHPLC-PDA Method for the Determination of R- and S-Ibuprofen

Supplementary Table S1 summarizes the validation results obtained for R-IBU and S-IBU in porcine plasma. Linear matrix-matched calibration curves with a range of 0.25–40 μg/mL for both enantiomers, were obtained. Good correlation between analyte concentrations and detected responses was observed for both enantiomers, with correlation coefficient (r) values ranging between 0.9949 and 0.9991 and goodness-of-fit coefficient (gof) values between 3.66 and 9.04%. The acceptance criteria for within- and between-run accuracy and precision were met for all drugs at the specified concentration levels (Supplementary Table S1). The LOQ was 0.25 μg/mL for both enantiomers. The calculated LOD values, corresponding with a signal/noise (S/N) ratio of 3, were 0.128 and 0.165 μg/mL for R-IBU and S-IBU, respectively. No carry-over was present as there was no analyte detected in the solvent sample injected after the highest calibrator. No interfering peaks could be detected in any of the blank samples at the retention time of the drugs, meaning the specificity of the method was demonstrated.

### Animals

All pigs survived the surgery and all double lumen jugular catheters were functional the day after surgery. Six out of 48 pigs had an obstructed jugular catheter after several days or accidentally removed the catheter due to scrubbing against the wall (two control pigs and four pigs in the IBU group, on day 6 or later, except for one 8-week-old control pig who removed its catheter already 1 day after surgery). If the catheter was obstructed or removed during the trial, no further blood samples were taken. Ibuprofen however, was still given via the stomach button to evaluate drug safety. The day after surgery, all stomach buttons, except for one 6-month-old control pig, were functional. During the trial, one 1-week-old piglet in the IBU group (day 9), two 8-week-old piglets in the IBU group (days 5 and 8), three 6–7-month-old pigs in the IBU group (days 5, 6, and 9) and two 6–7-month-old pigs in the control group (days 1 and 2) had a dysfunctional button after several days due to obstruction or loss of the button. This also led to exclusion of the animal from the trial. If the stomach button was obstructed, it was left in place as the pigs did not seem to experience any nuisance. If the button was removed, the resulting wound was cleaned, disinfected and bandaged. Only two 1-week-old and one 4-week-old piglet, all in the control group, showed more apathy and had a body temperature ≥ 40°C. These piglets were successfully treated with meloxicam.

No significant changes in body temperature were observed during the trial between treatment and control group or between the different age groups (Supplementary Figure S1). Similarly, no significant differences were observed in the WBC count over time for both control and treatment group. However, the 1-week-old piglets treated with ibuprofen had a significant lower amount of leukocytes compared to the control group (Supplementary Figure S1).

### Pharmacokinetics of R-, S-, and Total Ibuprofen

#### Total Ibuprofen

**FIGURE 3:**
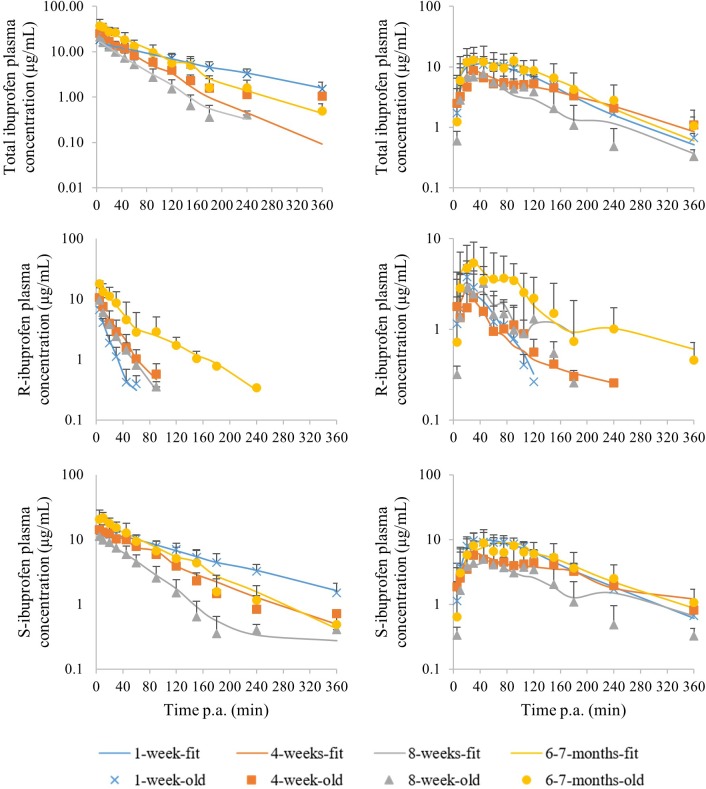
Median (+ standard deviation) plasma concentrations of total ibuprofen (top), R-ibuprofen (middle) and S-ibuprofen (bottom) after intravenous (left) and oral (right) administration of racemic ibuprofen (5 mg/kg BW) to each time 8 (4 ♂, 4 ♀) pigs aging 1 week (blue cross), 4 weeks (orange square), 8 weeks (gray triangle) and 6–7 months (yellow dot). The lines are the corresponding median model fits using the PK model.

The median plasma concentrations [+ standard deviation (SD)] and the corresponding median fit of total IBU after IV and PO administration are demonstrated in Figure 3. The PK parameters are given in Table 2. Both Cl and V_d_ showed an allometric relationship with BW with an allometric coefficient of 0.97 and 0.86, respectively (Supplementary Figure S2).

Significant sex differences were only observed at the age of 6–7 months for k_a_ after the first oral dose, C_max_ after 5 days of IBU dosing and AUC_0→3_ after IV administration and 5 days of oral dosing. Age did have a significant effect on all PK parameters.

#### R- and S-Ibuprofen

The median plasma concentrations (+SD) for R- and S-IBU after IV and PO administration can be found in Figure 3. The estimates of the PK parameters are given in Table 2. Supplementary Figure S2 demonstrates the allometric relationship between Cl and V_d_ of R- and S-IBU and BW. An allometric coefficient of 0.69, 0.79, 1.03, and 0.85 was estimated for Cl and V_d_ of R- and S-IBU, respectively.

After IV administration, C_0_ was higher in the 6–7 months old pigs compared to the other age groups for both enantiomers. This might be related to the V_d_. Hence, the V_d_ for R-IBU was the lowest in the 6–7 months old pigs, but a higher V_d_ in the 1-week-old pigs was observed compared to the 4-week-old pigs. Volume of distribution did not change during the first 8 weeks of life for S-IBU, but was significantly lower in the 6–7 months old pigs. No significant differences were observed between the 1-week- and 8-week-old pigs or the 4-week- and 8-week-old pigs regarding V_d_ of R-IBU. Clearance of S-IBU increased with age up until 8 weeks, after which it decreased. Clearance of R-IBU showed a sinusoidal course, namely higher in the 1-week- and 8-week-old pigs and lower in the 4-week- and 6–7-months-old pigs. The half-life of S-IBU was the highest in the 1-week-old pigs and did not change in the other age groups. However, T_1/2_ of R-IBU did show again the sinusoidal course similar but opposite to Cl of R-IBU, namely lowest in the 1-week- and 8-week-old pigs, highest in the 4-week- and 6–7-months old pigs. The AUC of S-IBU was always higher than that of R-IBU. The 1-week-old piglets had the lowest AUC for R-IBU and the AUC increased with age. The AUC of S-IBU in the 8-week-old pigs was lower compared to the other age groups.

After a single oral ibuprofen dose in the fasted state, no differences with age in T_1/2_, k_a_, T_max_, or C_max_ were observed for both enantiomers. Oral bioavailability only changed with age for R-IBU with the 4-week-old pigs having the lowest F compared to the other age groups. The AUC of R-IBU stayed the same during the first 8 weeks of life and was higher in the 6–7 months old pigs. The AUC of S-IBU on the other hand, was lower in the 8-week-old pigs compared to the 1-week- and 6–7-month-old pigs. Regarding significant differences between both enantiomers after oral dosing, only the 1-week-old piglets had a significant higher T_max_ and C_max_ for S-IBU compared to R-IBU. C_max_ of S-IBU at 4 weeks of age was also significant higher compared to that of R-IBU. And similar to the IV administration, the AUC of S-IBU was greater than the AUC of R-IBU in all age groups.

#### Multiple Oral Dosing of Ibuprofen

After 5 days dosing, few PK parameter estimates changed compared to the first oral dose (Table 3). C_max_ was lower for R-IBU the last day compared to the first oral dose and C_max,R_ was lower compared to C_max,S_ for the 1-week-, 4-week-, and 8-week-old pigs. A lower F for R-IBU (F_R_) was also observed. In the 4-week-old pigs, the AUC of both enantiomers was significantly lower after 5 days of IBU dosing.

The mean ratio of the AUC for R-IBU, S-IBU or total IBU after the first and last dose, as calculated according to Eq. 1, was lower than 1 for all four age groups. Results of the accumulation ratio can be found in Supplementary Table S2.

### Safety of Ibuprofen

Ibuprofen was well-tolerated in all pigs in every age group. During necropsy, no severe lesions could be observed in the stomach and consequently no significant differences were observed between the IBU group and the control group. Microscopic scoring revealed only significant differences between IBU and control group in the duodenum and jejunum for the 1-week-old pigs and in the antrum for the 4-week-old pigs. No significant histological changes were observed in the kidney. An overview of the mean sum of grading scores per tissue and group is given in Table 3.

The iohexol clearance (GFR) did not show any significant differences between the two administrations, namely at the start of the trial and after 5 days of IBU dosing, for all age groups. However, the eRPF (PAH clearance) was significantly higher at 4 weeks and 6–7 months of age. Boxplots of the results are given in Supplementary Figure S3.

The eRPF showed a good correlation with GFR which is reflected in almost identical allometric coefficients when Cl is plotted against GFR or eRPF (Supplementary Figure S2).

## Discussion

The current study aimed to evaluate developmental changes in pharmacokinetic parameters of R-, S-, and total ibuprofen in growing conventional pigs after single intravenous, single oral and multiple oral administration, as well as the drug’s safety profile.

**Table 2:** Pharmacokinetic parameters of total ibuprofen, R- and S-ibuprofen for intravenous (IV), first oral (PO) administration and oral administration after five consecutive treatment days.

Total ibuprofen												
	1-week-old			4-week-old			8-week-old			6–7-months-old		
	IV	PO (first bolus)	PO (after 5 days)	IV	PO (first bolus)	PO (after 5 days)	IV	PO (first bolus)	PO (after 5 days)	IV	PO (first bolus)	PO (after 5 days)
Cl (mL/(min^∗^kg)	2.3 (0.7)^a∗^			3.6 (1.1)^a^			5.6 (1.4)^b∗^			2.2 (0.7)^a^		
V_d_ (mL/kg)	308.9 (20.9)^a^			196.0 (58.0)^b^			293.4 (54.2)^a^			140.7 (32.6)^b^		
AUC_0→3 h_ (μg^∗^min/mL)	1804.1 (308.4)^a^	1575.8 (347.4)^ab^	1431.3 (422.1)^a^	1610.9 (437.3)^a^	1248.2 (535.1)^ab§^	707.0 (178.1)^b§^	949.1 (196.0)^b^	873.7 (400.9)^a^	718.3 (404.4)^b^	2436.0 (797.6)^c&^	1822.9 (900.8)^b^	1423.6 (1051.2)^ab&^
C_0_/C_max_ (μg/mL)	16.3 (1.1)^a^	13.8 (3.9)	12.0 (2.6)^ab^	28.0 (10.5)^b^	11.3 (7.4)	6.2 (4.7)^a^	17.5 (3.1)^a^	11.7 (4.8)	8.6 (4.4)^ab^	37.0 (7.7)^b^	17.2 (8.9)	16.8 (13.9)^b&^
T_max_ (min)		32.5 (20.0)^§^	60.0 (27.8)^§^		68.1 (61.3)	80.0 (36.1)		62.5 (40.4)	77.5 (45.9)		66.9 (48.3)	25 (13.2)
T_1/2_ (min)	96.8 (22.8)^a∗^	54.8 (19.4)^∗^	ns	38.5 (10.9)^b^	42.0 (38.1)	ns	37.6 (7.0)^b^	58.5 (86.8)	ns	46.8 (11.3)^b^	61.8 (49.0)	ns
k_a_ (1/min)		0.07 (0.08)^a^	0.04 (0.02)		0.01 (0.009)^b^	0.06 (0.06)		0.02 (0.009)^bc^	0.2 (0.4)		0.07 (0.07)^ac&^	0.02 (0.01)
F (%)		89.8 (23.7)	81.1 (25.0)		81.2 (39.2)	50.0 (20.7)		91.2 (31.7)	75.4 (35.7)		83.6 (46.5)	58.5 (35.8)
**R-ibuprofen**												
Cl (mL/(min^∗^kg)	10.6 (2.7)^a#^			5.3 (1.8)^b#^			7.5 (1.0)^ac∗#^			3.2 (2.2)^d^		
V_d_ (mL/kg)	329.9 (58.8)^a#^			259.5 (41.4)^b^			296.0 (64.7)^ab^			139.5 (33.1)^c^		
AUC_0→3 h_ (μg^∗^min/mL)	138.4 (28.1)^a#^	171.9 (73.4)^a#^	154.3 (62.0)^a#^	389.1 (176.4)^b#^	255.1 (119.2)^a§ #^	125.6 (51.0)^a§ #^	281.7 (48.7)^b#^	276.8 (114.3)^a#^	160.8 (78.1)^ab#^	801.8 (510.6)^c&#^	680.5 (295.3)^b#^	347.2 (315.8)^b&^
C_0_/C_max_ (μg/mL)	7.8 (1.4)^a#^	4.3 (1.7)^§ #^	2.2 (0.6)^a§ #^	9.9 (1.6)^ab^	3.7 (2.9)^§ #^	1.6 (1.9)^a§ #^	8.7 (1.5)^a^	4.8 (2.4)^§^	2.2 (1.3)^a§ #^	18.7 (3.4)^b^	7.5 (4.3)	6.7 (6.5)^b^
T_max_ (min)		17.5 (7.1)^#^	22.5 (12.5)^#^		43.1 (40.6)	58.6 (42.1)		59.4 (39.0)	60.8 (28.5)		53.8 (52.6)	18.0 (8.4)
T_1/2_ (min)	22.6 (5.5)^a∗#^	56.7 (53.5)^∗^	29.0 (12.6)^#^	36.2 (8.3)^b#^	58.3 (37.7)	56.5 (50.1)	27.7 (5.6)^a∗#^	84.9 (59.1)^∗#^	39.4 (23.1)	45.0 (27.2)^c∗^	286.7 (561.7)^∗^	29.6 (30.9)
K_a_ (1/min)	–	0.1 (0.1)	ns	–	0.08 (0.1)	ns	–	0.03 (0.02)	ns	–	0.1 (0.1)	ns
Cl R to S (ml/(min^∗^kg)	9.3 (2.1)^a^			4.6 (1.5)^b^			3.9 (0.7)^b^			1.6 (0.6)^c^		
F (%)		126.2 (46.8)^a^	110.4 (37.2)^a^		67.9 (33.8)^b^	36.6 (16.1)^b^		110.4 (55.2)^ab^	59.8 (32.9)^b^		102.2 (46.1)^ab§^	46.4 (36.0)^b§^
**S-ibuprofen**												
Cl (mL/(min^∗^kg)	1.9 (0.6)^a∗#^			2.6 (0.7)^a#^			4.9 (1.6)^b#^			2.1 (0.5)^a^		
V_d_ (mL/kg)	248.5 (26.2)^a∗#^			233.6 (90.6)^a^			275.4 (64.4)^a^			137.3 (36.0)^b^		
AUC_0→3 h_ (μg^∗^min/mL)	1671.2 (301.9)^a#^	1411.1 (309.6)^a#^	1307.1 (380.6)^a#^	1284.9 (372.6)^a#^	1019.7 (424.1)^ab§ #^	599.6 (146.2)^b§ #^	724.6 (199.8)^b#^	633.6 (293.1)^b#^	574.3 (348.6)^b#^	1655.7 (393.1)^a#^	1224.0 (591.6)^a#^	1088.1 (740.7)^ab^
C_0_/C_max_ (μg/mL)	10.1 (1.0)^a#^	11.1 (3.2)^#^	10.7 (2.3)^a#^	12.3 (4.9)^a^	7.9 (4.4)^#^	4.7 (2.8)^b#^	9.5 (2.0)^a^	7.1 (2.6)	6.7 (3.8)^ab#^	19.2 (4.6)^b^	9.9 (4.9)	10.5 (7.3)^a^
T_max_ (min)		48.8 (22.3)^#^	60.0 (11.3)^#^		87.5 (60.6)	84.3 (47.7)		70.0 (42.3)	77.5 (45.9)		83.8 (52.3)	32.0 (12.5)
T_1/2_ (min)	99.2 (23.6)^a∗#^	64.6 (18.5)^∗^	47.3 (16.9)^#^	61.4 (18.0)^b#^	64.1 (43.4)	54.8 (36.5)	41.0 (11.4)^b#^	34.7 (18.1)^#^	34.0 (15.0)	47.9 (14.4)^b^	38.0 (25.2)	26.1 (38.4)
K_a_ (1/min)		0.07 (0.07)	ns		0.06 (0.08)	ns		0.01 (0.009)	ns		0.01 (0.009)	ns
F (%)		86.9 (22.6)	80.5 (26.1)		83.5 (38.9)	54.2 (23.0)		86.6 (25.8)	79.0 (35.9)		79.3 (43.7)	64.7 (34.2)

*The mean and standard deviation are given for the 1-week-, 4-week-, 8-week- and 6–7-months-old pigs (each time 8 pigs, 4 ♂ and 4 ♀), respectively. Only clearance and volume of distribution after IV administration are given. Cl: clearance; V_d_: volume of distribution; AUC_0→∞_: area under the plasma concentration time curve from zero to infinity; C_0_: plasma concentration at time zero for the IV administration; C_max_: maximum plasma concentration for the PO administration; T_max_: time at which the C_max_ is reached; T_1/2_: elimination half-life; K_a_: absorption rate constant; Cl R to S: conversion rate of R-ibuprofen to S-ibuprofen; F: absolute oral bioavailability. Significant (p < 0.05) differences between the age groups for every PK parameter are annotated with different letters for all three administrations. If no significant differences were present, no annotations were made. If the PK parameter between the first and last day of oral dosing was not significantly different, this is demonstrated with ns. Significant differences within each age group between IV and PO (first bolus) administration are marked with an asterisk (^∗^). Similarly, significant differences between the first oral administration and the bolus after five consecutive treatment days are marked using §. Significant differences for the same PK parameter between R- and S-ibuprofen within that age category is marked with a #. Significant sex differences are marked with &.*

**Table 3:** The mean and standard deviation (SD) of the sum of macroscopic and histological scores per tissue for the ibuprofen (*n* = 8 per age group) and control group (*n* = 4 per age group) for the 1-week-, 4-week-, 8-week- and 6–7-months-old pigs.

	Duodenum	Jejunum	Ileum	Pars oesophagea	Antrum	Fundus	Macroscopic score
1-week-old pigs							
*Ibuprofen*	1.38 (0.52)^∗^	3.63 (2.88)^∗^	5.13 (0.99)	1.75 (2.19)	2.13 (1.13)	4.0 (1.77)	0.25 (0.46)
*Control*	4 (1.63)^∗^	1.25 (0.50)^∗^	5.25 (0.50)	1.75 (1.50)	3.50 (1.73)	5.0 (1.41)	0 (0)
4-week-old pigs							
*Ibuprofen*	3.50 (2.22)	3.63 (0)	3.13 (0)	3.75 (2.22)	2.75 (0.50)^∗^	0.75 (1.91)	2.13 (0.58)
*Control*	4.75 (2.22)	3.0 (0)	3.0 (0)	1.75 (2.22)	5.25 (0.50)^∗^	1.50 (1.91)	1.50 (0.58)
8-week-old pigs							
*Ibuprofen*	3.75 (0.71)	4.88 (1.64)	6 (0)	2.13 (1.55)	2.38 (1.69)	2.75 (1.16)	2.25 (1.04)
*Control*	4.0 (0.82)	4.25 (2.06)	6 (0)	1.25 (0.50)	2.75 (0.96)	2.75 (1.71)	3.0 (0.82)
6–7-months-old pigs							
*Ibuprofen*	3.25 (1.16)	3.38 (1.69)	6.38 (0.74)	4.50 (3.66)	2.88 (2.42)	2.13 (1.96)	1.75 (0.71)
*Control*	4.0 (0.82)	3.25 (1.89)	6.25 (0.50)	3.75 (3.77)	3.75 (1.71)	2.25 (1.89)	1.25 (0.50)

*Significant differences were determined with a Kruskal–Wallis test. Dunn‘s test was used for post hoc analysis. ^∗^Significant difference between ibuprofen and control group within that age group (p < 0.025).*

### Developmental Pharmacokinetics of Total Ibuprofen in Pigs

The absorption of IBU in the fasted state was significantly faster in the 1-week-old and 6–7 months old pigs compared to the other two age groups. In the 6–7 months old pigs, this is probably due to the greater contact surface area. In neonatal pigs however, the gastric pH is higher compared to older pigs. A higher pH would normally lead to less passive absorption in combination with a weak acid drug such as ibuprofen (pKa = 5.3) (Walthall et al., 2005). Nevertheless, since the 1-week-old piglets drank milk 1.5 h before administration, it is possible that the pH was lower leading to a faster absorption. The maturational changes in PK estimates will be discussed by means of the IV data. The Cl, when expressed per kg BW, increased with age up until 8 weeks of age, after which it decreased again (6–7 months old). Since IBU is known to be primarily metabolized in the liver, the maturation of CYP enzymes will be a defining factor for Cl as IBU is extensively metabolized by CYP2C8 and CYP2C9 in human (Rainsford, 2009). The homologs porcine CYP2C enzymes did increase with age in conventional pigs from a neonatal age till 8 weeks of age. Moreover, the amount of CYP2C35 in liver microsomes was lower in the 6–7 months old pigs compared to the 8-week-old pigs. This strengthens the suggestion that CYP2C35 might be involved in the biotransformation of IBU (Millecam et al., 2018). The Cl of IBU will also be influenced by the liver-to-body weight ratio (Rainsford, 2009). Millecam et al. (2018) suggested a log linear relationship between liver and body weight in conventional pigs from birth till puberty, with a maximum BW of 124 kg. In contrast, Hu (2015) observed a decreasing liver-to-body weight ratio after 5 weeks of age in Camborough-29 pigs. Since the oldest pigs in the current study all weighed more than 124 kg and the Cl is lower compared to the younger pigs, it is believed that the liver to body weight ratio would be much lower compared to these younger pigs. Hence, the observed non-linear relationship between non-weight-normalized Cl and weight do support these findings (Supplementary Figure S2). In human, the liver-to-body weight ratio also follows a non-linear curve with aging (‘t Jong, 2014). At last, both GFR and eRPF were significantly lower in the 6–7 months old pigs compared to the 8-week-old pigs. Since the metabolites of IBU are primarily renally excreted, these low renal physiological parameters will contribute to the lower Cl observed in these oldest pigs. The observed relationship between Cl and GFR and eRPF is then also almost linear (Supplementary Figure S2).

The V_d_, expressed per kg BW, showed a sinusoidal course with age, with the highest observed V_d_ in the 1-week- and 8-week-old pigs. These differences are probably due to the combination of maturation of the drug binding protein, albumin, and changes in the body composition. Neonatal pigs still have immature albumin levels which reach adult values around 1 year of age (Gasthuys et al., 2016). Low amounts of albumin leads thus to a higher free fraction of IBU and a higher V_d_. The body composition in pigs changes during development. Warnants et al. (2006) demonstrated that 4–10-week-old pigs had 10% fat, while 6-month-old pigs had > 20% fat. Although the log octanol-water partition coefficient is 3.97 for ibuprofen, a decreased V_d_ in obese adults compared to adults with a normal BW for IBU was observed and attributed to the body composition (Abernethy and Greenblatt, 1985). A lower V_d_ in 6–7 months old pigs with a high fat content is thus considered similar to human. Nevertheless, non-weight-normalized V_d_ did show an allometric relationship with weight (Supplementary Figure S2). The observed developmental differences in Cl and V_d_, which are not linearly related to BW, emphasize the importance of evaluating non-weight-normalized PK parameters.

Only limited sex differences in the 6–7-month-old pigs were noticed. After the first oral dose, k_a_ of total IBU was significantly higher in the females compared to the males. A clear hypothesis for this observation cannot be put forward. Next to that, the AUC_IV,0→3_ of total IBU was significantly higher in the males compared to the females. This might be due to the observed similar differences in AUC_IV,0→3_ for R-IBU. A higher AUC could suggest a slower R-to-S conversion. Nevertheless, no significant sex differences in Cl or Cl R to S were observed.

Several studies in children demonstrated a relationship between Cl and V_d_ and age (Supplementary Table S3). While Brown et al. (1992) observed a decreasing Cl and V_d_ with age, Har-Even et al. (2014) and Khalil et al. (2017) observed an increasing Cl and V_d_ with age/weight. The Cl in children was 10.3, 19.5, 32.8, and 81.3 mL/min for children aged 1, 6 months – 2, 2–6, and 6–16 years, respectively (Khalil et al., 2017). This is similar to the increasing whole body Cl observed in the current study, namely 6.8, 25.9, 111.1, and 307.5 mL/min for the 1-week-, 4-week-, 8-week-, and 6–7-month-old pigs, respectively. Similarly, V_d_ in the pediatric age groups as mentioned above increased from 1053.7 mL in the 1-month-old infants to 10314.2 mL in children aged 6–16 years. In pigs, V_d_ increased as well, namely 907.3 mL in the 1-week-old pigs toward 19527.1 mL in the 6–7-months old pigs. This limited available pediatric data might suggest that the juvenile pig could be a suitable animal model for the pediatric population. However, further research is required to evaluate allometric scaling or other *in silico* tools. Moreover, more thorough PK studies are warranted where all pediatric data are described in the most comprehensive way, since often only the mean parameters of a wide age range are provided.

### Enantiomeric Pharmacokinetics of Ibuprofen in the Growing Piglet

The developmental PK of R- and S-IBU showed great differences most likely attributed to their enantioselective behavior. Both enantiomers were rapidly absorbed in all age groups, but the C_max,S_ and T_max,S_ were always higher/later compared to R-IBU. This is probably due to the systemic stereochemical conversion of R-IBU to S-IBU. Pigs are able to perform chiral inversion, as was shown after administration of the pure R-enantiomer of ketoprofen (Neirinckx et al., 2011b). The rate of stereochemical conversion of R-IBU decreased with age and was the highest in the 1-week-old piglets (Table 2). Since no urine was collected, it was not possible to determine the fraction of the dose converted. In human adults, the conversion rate was estimated to be 0.53–0.82 mL/min/kg with a total fraction of 0.48 to 0.68 of the dose being converted (Rudy et al., 1991; Tan et al., 2003). In the pediatric population, very limited data is available regarding the conversion of IBU. Gregoire et al. (2008) estimated that 17% of R-IBU was converted to S-IBU in premature newborn infants. Rey et al. (1994) found the plasma concentrations of S-IBU always to be smaller than those of R-IBU probably due to impaired conversion or higher S-IBU clearance. It should be noted however that these infants were treated with IBU during surgery recovery, meaning that the after-effects of the anesthesia could possibly have affected the PK of IBU. Generation of more pediatric data is warranted to obtain a full developmental profile of the stereochemical conversion of R- to S-IBU since the results of Rey et al. (1994) are currently generalized for the complete pediatric population although it only covers infants (Rey et al., 1994; Rainsford, 2009).

No significant differences could be found between R-IBU and S-IBU regarding F. While F_S_ did not change with age, F_R_ was significantly lower at 4 weeks of age compared to the 1-week-old piglets. This might be an indication of pre-systemic conversion. Nevertheless, since no differences in PK parameters between IV and PO administration at 4 weeks of age were observed, it is highly doubtful if pre-systemic conversion does actually occur. The 4-week-old pigs were weaned at arrival at the test facility and this could have had an influence on F_R_. It is known that weaning activates several immune and inflammatory responses, which are likely a cause of small intestine atrophy (Bomba et al., 2014; Cao et al., 2018). Consequently, this might lead to enantioselective absorption with a preference for the S-enantiomer or faster pre-systemic elimination of R-IBU. Enantioselective absorption, however, has not yet been reported in literature.

The Cl of R- and S-IBU changed differently during the first 4 weeks of life. While Cl of R-IBU (Cl_R_) decreased, Cl of S-IBU (Cl_S_) increased during these first 4 weeks. These developmental changes in porcine Cl are similar to pediatric data generated by Dong et al. (2000), where a decreasing Cl_R_ with age and a higher weight normalized Cl_R_ in children (2–13 years) compared to adults was found. The S-enantiomer showed no correlation with age in these children.

Both enantiomers had the lowest V_d_ at 6–7 months which could be attributed to the body composition as discussed above. After IV administration, V_R_ was higher compared to V_S_ in the 1-week-old piglets (329.9 versus 248.5 mL/kg respectively). Neonatal pigs still have immature albumin concentrations, making them more subject to differences in enantioselective protein binding. In human, the protein binding is competitive and enantioselective, with a higher affinity of R-IBU for albumin compared to S-IBU. This leads to a higher free fraction of S-IBU and consequently a higher V_S_ in human. The results in the neonatal pigs however, suggest otherwise, namely higher albumin affinity for S-IBU (Hao et al., 2005). This hypothesis should be verified with protein binding experiments using both racemic ibuprofen and the individual enantiomers.

The enantiomeric differences in T_1/2_ were also comparable to human. In premature new-born infants, T_1/2,S_ was found to be longer than T_1/2,R_ (2,058 vs. 498 min on post-natal day 1, Supplementary Table S3), which was also observed in the 1-week-old piglets (99.2 vs. 22.6 min) (Gregoire et al., 2008). The differences in porcine T_1/2_ became smaller with aging, as was also observed in human by Kelley et al. (1992), Dong et al. (2000), and Tan et al. (2003). Rey et al. (1994) on the other hand, found no enantiomeric differences in T_1/2_.

While the PK of the IBU enantiomers in the growing pig does show some similarities with the available human data, thorough comparison is impossible due to the lack of extensive PK studies evaluating both enantiomers in the complete pediatric population. Further research is warranted.

### Multiple Oral Dosing

Consecutive oral dosing for 5 days did alter the enantioselective PK characteristics in growing piglets although no accumulation was observed. In children aged 4–11 years, also no IBU accumulation occurred after five oral doses of an IBU-pseudoephedrine suspension every 6 h. However, the PK characteristics were only determined after the fifth dose (Gelotte et al., 2010). In humans, the absorption of IBU tablets is believed to be determined by gastric emptying and the gastro-intestinal transit time (Neirinckx et al., 2011a; Koenigsknecht et al., 2017). The current study used a suspension which was apparently not influenced by the fed state, as demonstrated by the absent differences in T_max_ or k_a_ between the first and last oral administration of the multiple oral dosing study. However, C_max,R_ and F_R_ were decreased, except for the 1-week-old pigs(F_R_) and 6–7 months old pigs (C_max,R_), which could mean that pre-systemic conversion or elimination occurred upon multiple dosing. Unfortunately, no extensive similar human PK data are available. Most human PK trials are single dose only or the PK studies were only performed at the start or end of the trial when multiple dosing was done, but not on both occasions as in the current study.

### Safety Profile of Ibuprofen

Since only the duodenum and jejunum of the 1-week-old IBU group showed a higher inflammatory response, while the other significant higher responses were observed in the control group, IBU was considered to be safe to administer to pigs from 1-week-old till 6–7-months of age. This is consistent with the low incidence of adverse events observed in children (Rainsford, 2009; de Martino et al., 2017; Ziesenitz et al., 2017). Regarding renal safety, elevated eRPF was observed in the 4-week- and 6–7-months-old pigs, but no differences in GFR were observed. This is in contrast with the results from Junot et al. (2017) where both a decreased GFR and renal blood flow were observed after administration of ketoprofen to pigs weighing 25–32 kg. It would be expected that NSAIDs such as IBU, decrease GFR and eRPF due to their inhibitory effect on the formation of vasodilatating prostaglandins (Kim, 2008). However, since pigs are able to acetylate PAH, this route of elimination needs to be taken into account when determining the true RPF (Troncy et al., 1997). The eRPF determined in the current study represents both renal and metabolic clearance. The increased eRPF could be attributed to an increased acetylation capacity instead of an IBU-related vasodilatation which would be contradictory. In conclusion, 5 days dosing of IBU did not alter the renal function of the piglets.

## Conclusion

The developmental and enantioselective PK of IBU in the growing piglet was demonstrated. Multiple oral dosing did affect some PK parameters, decreased the bioavailability of R-IBU and was shown to be safe. Age did affect the rate of stereochemical conversion. The limited human PK data available showed a similar increase in Cl and V_d_ of total ibuprofen as observed in the current study, suggesting the conventional pig as a suitable animal model to evaluate ibuprofen and possibly other NSAIDs. Nevertheless, more comprehensive pediatric data regarding the IBU enantiomers is warranted.

## Ethics Statement

The current study was approved by the ethical committee of the Faculties of Veterinary Medicine and Bioscience Engineering of Ghent University (EC2016/105). Care and use of the animals were in full compliance with the national and European legislation on animal welfare and ethics (Flemisch Government 2017) and (Eur-Lex, 2010).

## Author Contributions

JM, JVW, EG, SC, and MD contributed conception and design of the study. JM performed the animal trials, bioanalytical, histological, pharmacokinetic, statistical analysis, and wrote the first draft of the manuscript. TvB, SS, GA, and AM performed surgical procedures necessary for this study. KC aided in the histological analysis. MD and RG aided in the pharmacokinetic analysis. All authors contributed to manuscript revision, read and approved the submitted version.

## Conflict of Interest Statement

The authors declare that the research was conducted in the absence of any commercial or financial relationships that could be construed as a potential conflict of interest.
