# Supplementary material for: Developmental Pharmacokinetics and Safety of Ibuprofen and Its Enantiomers in the Conventional Pig as Potential Pediatric Animal Model
Source: Front Pharmacol. 2019 May 9;10:505. doi: 10.3389/fphar.2019.00505 (PMC6521589; doi:10.3389/fphar.2019.00505)
Supplement: Supplementary file 1 [file Table_1.DOCX]

**Supplemental Data**

# 1. Materials and Methods

- 1. **Determination of R- and S-ibuprofen in porcine plasma**

Plasma concentrations of R- and S-ibuprofen were quantified by an in-house validated UHPLC method with a photodiode array detector (PDA). Stock solutions of 1 mg/mL of R-ibuprofen (Sanbio B.V., Uden, The Netherlands), S-ibuprofen (Sanbio B.V.,) and the internal standard (IS) fenoprofen (Sigma Aldrich) were made in methanol and stored at ≤ -15°C. Working solutions with a mix of R- and S-ibuprofen were made at different concentrations in methanol and also stored at ≤ -15°C. A working solution of 100 µg/mL in methanol was prepared for the IS and stored at ≤ -15°C. All solvents used were UPLC grade. The sample preparation was a combination of liquid-liquid extraction with ethyl acetate and derivatization with L-leucinamide (L-leucinamide hydrochloride, Sigma Aldrich). In short, after spiking 250 µL of plasma with 25 µL of IS working solution, 200 µL of HCl 1N (p.a. grade, Thermo Fisher Scientific, Merelbeke, Belgium) in water and 5 mL of ethyl acetate (p.a. grade, Merck, Darmstadt, Germany) were added. After 15 min gentle rolling, the samples were centrifuged for 5 min at 3,724 *g* (Beckman Coulter Allegra X-15R, Brea, California, USA) and the organic phase was evaporated under nitrogen at 40°C. Next, the derivatization was started according to Plessers et al. (2015) with the addition of 100 µL of 50 mM triethylamine (Sigma Aldrich) in acetonitrile (ACN) followed by 50 µL of 60 mM ethylchloroformate (Sigma Aldrich) in ACN and 50 µL of a mixture of 1 M L-leucinamide and 1 M triethylamine (1:1) in ACN (Plessers et al. 2015). Before and after addition of L-leucinamide, an equilibration time of 3 min was respected. After the derivatization, a second liquid-liquid extraction was performed by adding 200 µL of HCl 1N and 3 mL of ethyl acetate, followed by 5 min shaking on a rotating shaker at 75 rpm (Trayster digital, IKA®, Staufen, Germany). After pulse centrifugation for 20 s, the organic phase was again evaporated and dissolved in 200 µL of 95/5 water/ACN. The sample was finally filtered through a Millex-GV PVDF filter (0.22 µm, Merck) and brought in a conical autosampler vial.

A 5 µL aliquot of the sample was injected onto an Acquity UHPLC PDA system (Waters, Zellik, Belgium). Chromatographic separation was achieved on an Acquity UPLC CSH Phenyl-Hexyl column (150 mm x 2.1 mm i.d., dp: 1.7 µm) in combination with a pre-column of the same type (5 mm x 2.1 mm i.d., dp: 1.7 µm), both from Waters. The mobile phase A consisted of water and the mobile phase B consisted of ACN. The following gradient was applied: 0-2 min (70% A, 30% B), 2-2.5 min (linear gradient to 53% B), 2.5-9 min (47% A, 53% B), 9-9.2 (linear gradient to 70% A) and 9.2-15 (70% A, 30% B). The flow rate was 0.2 mL/min and the detector was set at 223 nm. The temperature of the autosampler and column were set at 6°C and 22°C, respectively. Retention times were 13.2, 13.6, 14.0 and 14.2 min for R-, S-fenoprofen and R-, S-ibuprofen respectively. The R enantiomer of fenoprofen was used as IS for quantification. A matrix-matched calibration curve for each enantiomer was made with concentrations ranging from 0.25 µg/mL to 40 µg/mL.

The described analytical method for the determination of ibuprofen enantiomers in porcine plasma was validated according to European and international guidelines and recommendations (Knecht and Stork 1974, US Department of Health and Human Services 2015, European Commission 2002). The following parameters were evaluated: linearity (correlation coefficient, r and goodness-of-fit coefficient, gof), within-run and between-run precision and accuracy, limit of quantification (LOQ), limit of detection (LOD), carry-over and stability of the analytes of interest in sample extracts during storage in the autosampler (temperature: 6°C).

- 1. **Determination of iohexol and PAH in porcine plasma**

Iohexol and PAH were measured in porcine plasma according to a multi-UHPLC-MS/MS method developed and validated by (Dhondt et al. 2019). Iohexol-d5 (Alsachim, Illkirch Graffenstaden, France) and p-aminobenzoic acid (PABA, Sigma Aldrich) were used as IS. In short, sample preparation consisted of deproteinization of 100 µL plasma using 1 mL of methanol. After centrifugation for 15 min at 18,533 *g* and 4°C, samples were evaporated under nitrogen at 40°C and redissolved in 250 µL of UPLC water. Calibration curves were prepared in a concentration range from 0.25 – 90 µg/mL for both compounds. The LOQ was 0.25 µg/mL for both compounds and the LOD was 84 and 3.8 ng/mL for iohexol and PAH, respectively. Chromatographic separation was achieved on a Hypersil Gold aQ column (100 mm x 2.1 mm i.d., dp: 1.9 µm) in combination with a 2.1 mm ID filter cartridge (Thermo Scientific) using gradient elution. Mobile phase A consisted of 0.1% formic acid (Sigma Aldrich) in water and mobile phase B consisted of 0.1% formic acid in methanol and the flow rate was set at 0.3 mL/min. The MS/MS instrument (Quattro Premier XE, Waters) was operated in the positive electrospray ionization mode. MS/MS acquisition was performed in the selected reaction monitoring (SRM) mode. The following traces *m/z* traces were monitored for quantification and identification respectively: iohexol *m/z* 822 > 804 and *m/z* 822 > 731.2; iohexol-d5 *m/z* 827 > 809 and *m/z* 827 > 736.1; PAH *m/z* 195.1 > 119.9 and *m/z* 195.1 > 91.9; PABA *m/z* 137.8 > 77.2 and *m/z* 137.8 > 65.1.

1. **Results**

**Supplemental Table S1**: Results of the evaluation of linearity, limit of quantification, limit of detection and within-day and between-day precision and accuracy for R-ibuprofen and S-ibuprofen in porcine plasma.

| Analyte | calibration range (µg/mL) | gof (%) | r | LOQ (µg/mL) | LOD (µg/mL) |
| --- | --- | --- | --- | --- | --- |
| R-IBU | 0.25 – 40 | 6.07 | 0.997 | 0.25 | 0.128 |
| S-IBU | 0.25 – 40 | 4.84 | 0.998 | 0.25 | 0.165 |
|  |  | **Within-day (n = 6)** | | **Between-day (n = 3x3)** | |
|  | theoretical concentration (µg/mL) | accuracy (%) | precision (RSD, %) | accuracy (%) | precision (RSD, %) |
| R-IBU | 0.5 | -7.6 | 5.5 | -1.8 | 5.4 |
|  | 5 | -0.3 | 7.1 | 0.9 | 7.1 |
|  | 20 | -2.0 | 4.4 | 2.2 | 3.6 |
| S-IBU | 0.5 | -3.0 | 5.9 | 0.1 | 5.5 |
|  | 5 | 2.3 | 1.9 | 1.8 | 2.7 |
|  | 20 | 3.8 | 4.0 | 5.9 | 3.0 |
| R-IBU: R-ibuprofen; S-IBU: S-ibuprofen; gof: goodness-of-fit coefficient; r: correlation coefficient; LOQ: limit of quantification; LOD: limit of detection; RSD: relative standard deviation.  Acceptance criteria for linearity: r > 0.99 and g < 10%  Acceptance criteria for within- and between-day accuracy: -20% to +10%  Acceptance criteria for within-day precision (RSD_max_): 10%  Acceptance criteria for between-day precision (RSD_max_): 0.5 µg/mL: 17.8%; 5 µg/mL: 12.6% and 20 µg/mL: 10.2% | | | | | |


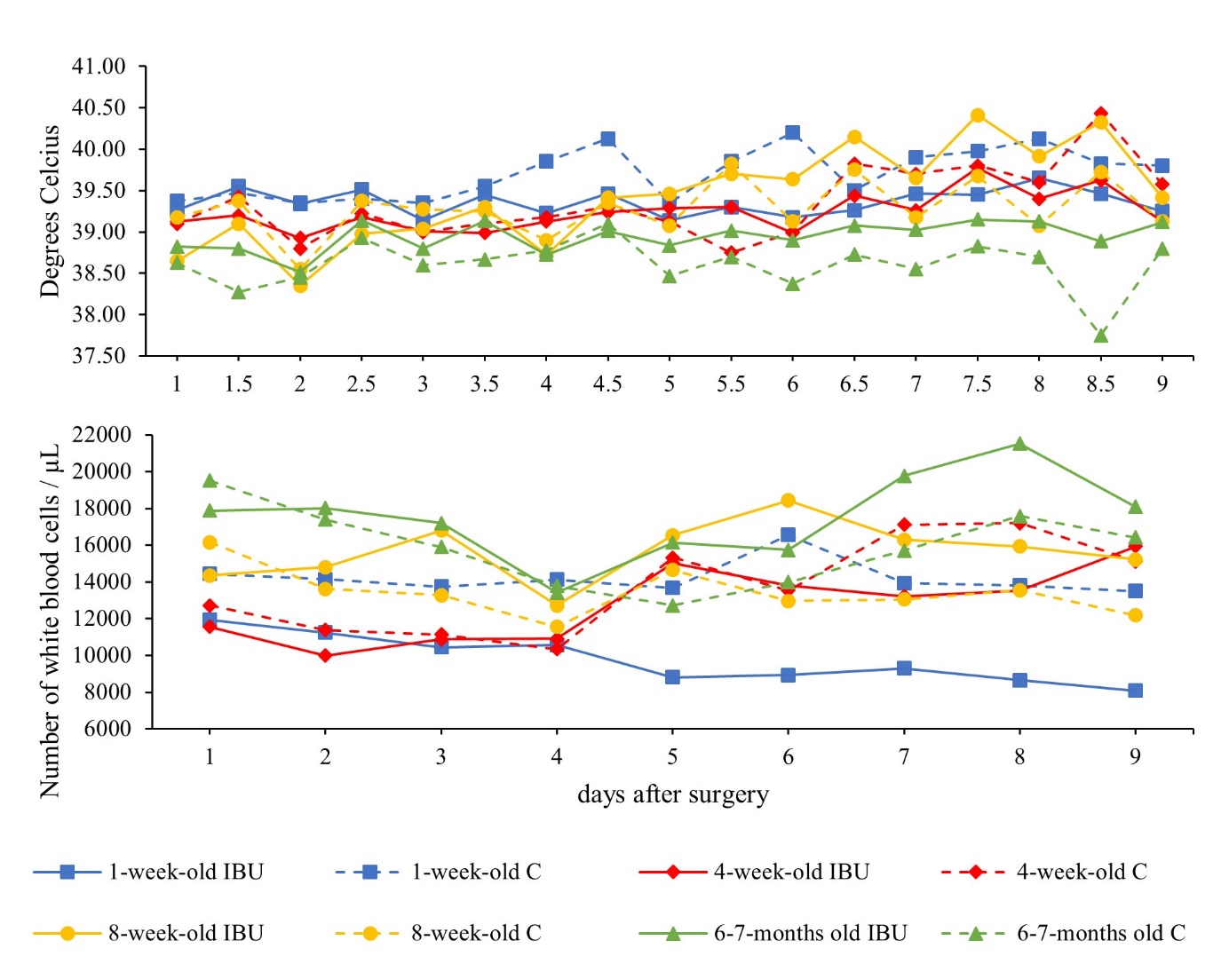


**Supplemental Figure S1**: On top, the mean body temperature and below the mean number of white blood cells for the control pigs (4 pigs per age groups, C, dotted lines) and pigs receiving ibuprofen (8 pigs per age groups, IBU, full lines) at 1-week (blue square), 4-weeks (red diamond), 8-weeks (yellow dot) and 6-7 months (green triangle) of age. Measurements started the day after surgical placement of a double lumen jugular catheter and a gastrostomy button.

**Supplemental Figure S2**: Illustration of the allometric relationships between clearance (Cl) and volume of distribution (V_d_) on the one hand and weight on the other hand of total ibuprofen (IBU), R-ibuprofen (R-IBU) and S-ibuprofen (S-IBU); between Cl and glomerular filtration rate (GFR) and estimated renal plasma flow (eRPF) of total IBU, R-IBU and S-IBU; and between GFR, eRPF and weight of the pigs. All parameters were obtained after IV administration of 5 mg/kg BW racemic ibuprofen in each time 8 pigs (4 male, 4 female) ageing 1 week, 4 weeks, 8 weeks and 6-7 months.

**Supplemental Table S2**: Overview of the mean (standard deviation) accumulation ratio for total ibuprofen (IBU), R-ibuprofen (R-IBU) and S-ibuprofen (S-IBU) after three oral doses of 5 mg/kg BW racemic ibuprofen per day for five consecutive days.

|  | Total IBU | R-IBU | S-IBU |
| --- | --- | --- | --- |
| 1 week old (n = 8) | 0.97 (0.40) | 1.08 (0.70) | 0.98 (0.37) |
| 4 weeks old (n = 7) | 0.70 (0.19) | 0.67 (0.28) | 0.72 (0.22) |
| 8 weeks old (n = 8) | 0.89 (0.35) | 0.64 (0.31) | 0.98 (0.39) |
| 6-7 months old (n = 6) | 0.83 (0.50) | 0.52 (0.31) | 0.97 (0.54) |

(A)


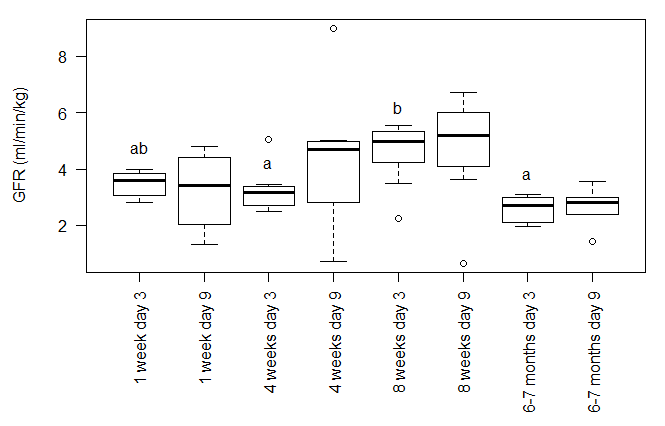


age and experimental day

(B)


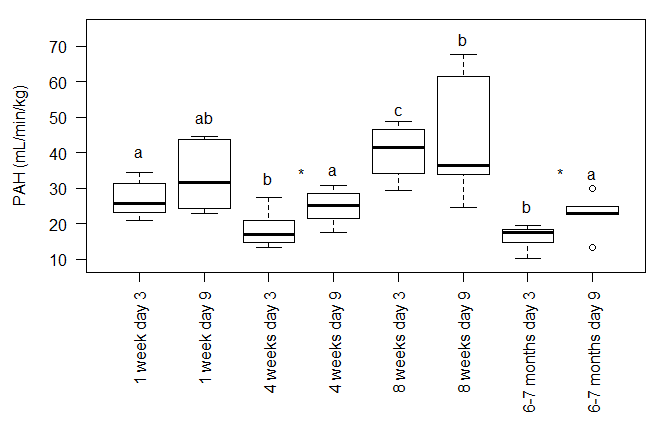


age and experimental day

**Supplemental Figure S3**: Boxplots of the glomerular filtration rate (GFR, A) and the estimated renal plasma flow (eRPF, B) in all four age groups (each time 8 pigs, 4 ♂ and 4 ♀) on day 3 (IV administration) and day 9 (last day of PO administration). The boxplots give the median, 25^th^ and 75^th^ percentiles. The upper and lower whisker extends from the hinge to the largest/smallest value respectively no further than 1.5 times the interquartile range. Data beyond the end of the whiskers are outliers and plotted individually. Significant differences (p < 0.05) between start and end of the trial are indicated with an asterisk (*). Significant differences between age groups are annotated with a different letter.

**Supplemental Table S3:** Overview of the available pharmacokinetic (PK) studies after administration of racemic ibuprofen in children and adults. The mean PK parameters are given.

| Reference | No. of patients | Age | Clinical setting | Dose and route | Mean Pharmacokinetic parameters | Main results/ conclusion/others |
| --- | --- | --- | --- | --- | --- | --- |
| (a) Studies investigating the pharmacokinetics of the enantiomers after racemic ibuprofen dosing. | | | | | | |
| (Gregoire et al. 2008) | 108 | GA 24-30.7 weeks; PND 0 | PDA | 10/5/5 mg/kg IV | Cl_S_ 0.058 mL/min/kg  AUC_S_ 50,100 µg*min/mL  T_1/2,S_ 2,058 min  Cl_R_ 0.43 mL/min/kg  AUC_R_ 5,880 µg*min/mL  T_1/2,R_ 498 min | 17% of R-ibuprofen is estimated to be converted to S-ibuprofen. |
|  |  | GA 24-30.7 weeks; PND 3 |  |  | Cl_S_ 0.058 mL/min/kg  AUC_S_ 44,040 µg*min/mL  T_1/2,S_ 2,058 min  Cl_R_ 2.8 mL/min/kg  AUC_R_ 900 µg*min/mL  T_1/2,R_ 78 min |  |
|  |  | GA 24-30.7 weeks; PND 8 |  |  | Cl_S_ 0.058 mL/min/kg  AUC_S_ 43,380 µg*min/mL  T_1/2,S_ 2,058 min  Cl_R_ 6.75 mL/min/kg  AUC_R_ 360 µg*min/mL  T_1/2,R_ 30 min |  |
| (Rey et al. 1994) | 11 | 6-18 months | During recovery of minor genito-urinary surgery | Mean 7.6 ± 0.3 mg/kg oral syrup | C_max,S_ 9.7 µg/mL  AUC_S_ 1,890 µg*min/mL  T_1/2,S_ 96 min  C_max,R_ 11.8 µg/mL  AUC_R_ 2,196 µg*min/mL  T_1/2,R_ 90 min  Cl/F_R_ 2 mL/min/kg  V_d_/F_R_ 300 mL/kg | No significant correlation between PK parameters and age.  Plasma concentrations S < R due to impaired conversion or higher clearance of S. |
| (Kelley et al. 1992) | 17 | 6 months – 11 years | Febrile | 6 mg/kg oral liquid | C_max,S_ 13.86 µg/mL  T_max,S_ 66.4 min  T_1/2,S_ 138.6 min  K_a,S_ 0.034 /min  Cl/F_S_ 1.24 mL/min/kg  V_d_/F_S_ 248 mL/kg  C_max,R_ 13.39 µg/mL  T_max,R_ 44.7 min  T_1/2,R_ 88.2 min  K_a,R_ 0.049 /min  Cl/F_R_ 1.24 mL/min/kg  V_d_/F_R_ 158 mL/kg |  |
| (Dong et al. 2000) | 38 | 2-13 years | Cystic fibrosis | 20 mg/kg as suspension or tablet | C_max,S_ 41.1 µg/mL  AUC_S_ 7,200 µg*min/mL  T_1/2,S_ 99.6 min  Cl/F_S_ 1.62 mL/min/kg  V_d_/F_S_ 204 mL/kg  Cmax_R_ 33.9 µg/mL  AUC_R_ 3,534 µg*min/mL  T_1/2,R_ 85.2 min  Cl/F_R_ 3.3 mL/min/kg  V_d_/F_R_ 364 mL/kg | Different formulations had no significant impact on PK parameters. No significant gender differences. Significant decreasing Cl/F_R_ with age.  Weight normalized Cl/F_R_ in CF children significantly higher than adults. |
| (Rudy et al. 1991) | 8 | Adults (19-41 years) | Healthy | 800 mg gelatin capsule | Cl/F_S_ 1.25 mL/min/kg  Cl R to S 0.82 mL/min/kg  Cl/F_R_ 0.80 mL/min/kg | 48-66% of R-ibuprofen is estimated to undergo conversion. |
| (Tan et al. 2003) | 12 | Adults (20-29 years) | Healthy | 400 mg tablet | C_max,S_ 17.5 µg/mL  T_max,S_ 156 min  T_1/2,S_ 138 min  AUC_S_ 4,758 µg*min/mL  Cl/F_S_ 1.0 mL/min/kg  V_d_/F_S_ 202.7 mL/kg  fu_S_ 0.47%  C_max,R_ 16.3 µg/mL  T_max,R_ 144 min  T_1/2,R_ 84 min  AUC_R_ 3,636 µg*min/mL  Cl R to S 0.53 mL/min/kg  Cl/F_R_ 0.25 mL/min/kg  V_d_/F_R_ 116.7 mL/kg  fu_R_ 0.23% | 68% of R-ibuprofen is estimated to undergo conversion. |
| Reference | No. of patients | Age | Clinical setting | Dose and route | Mean Pharmacokinetic parameters | Main results/ conclusion/others |
| (b) Studies investigating the pharmacokinetics of total ibuprofen after racemic ibuprofen dosing. | | | | | | |
| (Brown et al. 1992) | 38 | 3 months – 2.5 years | Febrile | 5 mg/kg liquid | C_max_ 19.03 µg/mL  T_max_ 96 min  T_1/2_ 99 min  K_a_ 0.14 /min  AUC 3,433.8 µg*min/mL  Cl/F 1.77 mL/min/kg  V_d_/F 221 mL/kg | Significant effect of age on AUC, Cl/F and V/F. |
|  |  | 2.5-12 years |  |  | C_max_ 19.03 µg/mL  T_max_ 96 min  T_1/2_ 99 min  K_a_ 0.14 /min  AUC 5,296.8 µg*min/mL  Cl/F 1.12 mL/min/kg  V_d_/F 133 mL/kg |  |
|  | 46 | 3 months – 2.5 years |  | 10 mg/kg liquid | C_max_ 34.35 µg/mL  T_max_ 92.4 min  T_1/2_ 88.8 min  K_a_ 0.11 /min  AUC 6,148.8 µg*min/mL  Cl/F 1.95 mL/min/kg  V_d_/F 258 mL/kg |  |
|  |  | 2.5-12 years |  |  | C_max_ 34.35 µg/mL  T_max_ 92.4 min  T_1/2_ 88.8 min  K_a_ 0.11 /min  AUC 7,558.8 µg*min/mL  Cl/F 1.43 mL/min/kg  V_d_/F 186 mL/kg |  |
| (Gelotte et al. 2010) | 24 | 4-11 years | Healthy | 7.5 mg/kg suspension  5 doses every 6 hours | C_max_ 32.0 µg/mL  T_max_ 58.2 min  T_1/2_ 78 min  K_a_ 0.031 /min  AUC_0🡪6h_ 5,958 µg*min/mL  Cl/F 1.29 mL/min/kg  V_d_/F 147 mL/kg | No drug accumulation. |
| (Har-Even et al. 2014) | 28 | 3 months – 15 years | Suspicion of sepsis, 14 diagnosed with meningitis | Mean 9.5 ± 1.6 mg/kg | K_a_ 0.009 /min  Cl/F 1.55 mL/min/kg  V_d_/F 39 mL/kg  T_lag_ 25 min | Significant effect of weight on K_a_ and Cl/F. Great variability in V/F and T_lag_. |
| (Khalil et al. 2017) | 1 | 1 month | Febrile | 10 mg/kg IV infusion over 10 min | C_max_ 49.8 µg/mL  T_max_ 10 min  T_1/2_ 108 min  AUC_0🡪4_ 4,148.4 µg*min/mL  Cl 10.3 mL/min  V_d_ 1,053.7 mL | Cl and V increased with age, nevertheless no weight-normalised data was available. |
|  | 5 | 6 months – 2 years |  |  | C_max_ 59.2 µg/mL  T_max_ 14 min  T_1/2_ 106.8 min  AUC_0🡪4_ 4,255.2 µg*min/mL  Cl 19.5 mL/min  V_d_ 2,805.7 mL |  |
|  | 12 | 2-6 years |  |  | C_max_ 64.2 µg/mL  T_max_ 18.5 min  T_1/2_ 88.8 min  AUC_0🡪4_ 4,815 µg*min/mL  Cl 32.8 mL/min  V_d_ 3,695.8 mL |  |
|  | 25 | 6-16 years |  |  | C_max_ 61.9 µg/mL  T_max_ 12.7 min  T_1/2_ 93 min  AUC_0🡪4_ 5,143.8 µg*min/mL  Cl 81.3 mL/min  V_d_ 10,314.2 mL |  |
| Cl/F: clearance; V/F: volume of distribution; AUC_0→t_: area under the plasma concentration time curve from zero to time mentioned in hour; C_max_: maximum plasma concentration; T_max_: time at which the C_max_ is reached; T_1/2_: elimination half-life; Ka: absorption rate constant; Cl R to S: conversion rate of R-ibuprofen to S-ibuprofen; T_lag_: lag time; GA: gestational age; PND: post-natal days; PDA: percutaneous ductus arteriosus; IV: intravenous; fu: fraction unbound | | | | | | |

Brown, R. D., J. T. Wilson, G. L. Kearns, V. F. Eichler, V. A. Johnson, and K. M. Bertrand. 1992. "Single-dose pharmacokinetics of ibuprofen and acetaminophen in febrile children." *J Clin Pharmacol* 32 (3):231-41.

Dhondt, Laura, Siska Croubels, Pieter De Cock, Pieter De Paepe, Siegrid De Baere and Mathias Devreese. 2019. "Development and validation of an ultra-high performance liquid chromatography-tandem mass spectrometry method for the simultaneous determination of iohexol, p-aminohippuric acid and creatinine in porcine and broiler chicken plasma." *Journal of Chromatography B*, in press.

Dong, J. Q., L. Ni, C. S. Scott, G. Z. Retsch-Bogart, and P. C. Smith. 2000. "Pharmacokinetics of ibuprofen enantiomers in children with cystic fibrosis." *J Clin Pharmacol* 40 (8):861-8.

European Commission. 2002. "2002/657/EC, implementing Council Directive 96/23/EC concerning the performances of analytical methods and interpretation of results."

Gelotte, C. K., M. J. Prior, C. Pendley, B. Zimmerman, and B. J. Lavins. 2010. "Multiple-dose pharmacokinetics and safety of an ibuprofen-pseudoephedrine cold suspension in children." *Clin Pediatr (Phila)* 49 (7):678-85. doi: 10.1177/0009922810363153.

Gregoire, N., L. Desfrere, J. C. Roze, Y. Kibleur, and P. Koehne. 2008. "Population pharmacokinetic analysis of Ibuprofen enantiomers in preterm newborn infants." *J Clin Pharmacol* 48 (12):1460-8. doi: 10.1177/0091270008323752.

Har-Even, R., D. Stepensky, M. Britzi, S. Soback, A. B. Chaim, N. Brandriss, M. Goldman, M. Berkovitch, and E. Kozer. 2014. "Plasma and cerebrospinal fluid concentrations of ibuprofen in pediatric patients and antipyretic effect: Pharmacokinetic-pharmacodynamic modeling analysis." *J Clin Pharmacol* 54 (9):1023-30. doi: 10.1002/jcph.307.

Kelley, M. T., P. D. Walson, J. H. Edge, S. Cox, and M. E. Mortensen. 1992. "Pharmacokinetics and pharmacodynamics of ibuprofen isomers and acetaminophen in febrile children." *Clin Pharmacol Ther* 52 (2):181-9.

Khalil, S. N., B. J. Hahn, C. E. Chumpitazi, A. D. Rock, B. A. Kaelin, and C. G. Macias. 2017. "A multicenter, randomized, open-label, active-comparator trial to determine the efficacy, safety, and pharmacokinetics of intravenous ibuprofen for treatment of fever in hospitalized pediatric patients." *BMC Pediatr* 17 (1):42. doi: 10.1186/s12887-017-0795-y.

Knecht, J, and G Stork. 1974. "Percentage and logarithmic procedures for calculation of calibration curves." *Fresenius Zeitschrift Fur Analytische Chemie* 270 (2):97-99. doi: 10.1007/BF00434058.

Plessers, E., A. Watteyn, H. Wyns, B. Pardon, S. De Baere, P. De Backer, and S. Croubels. 2015. "Enantioselective pharmacokinetics of ketoprofen in calves after intramuscular administration of a racemic mixture." *J Vet Pharmacol Ther* 38 (4):410-3. doi: 10.1111/jvp.12186.

Rey, E., A. Pariente-Khayat, L. Gouyet, F. Vauzelle-Kervroëdan, G. Pons, P. D'Athis, M. C. Dubois, I. Murat, C. Lassale, and M. Goehrs. 1994. "Stereoselective disposition of ibuprofen enantiomers in infants." *Br J Clin Pharmacol* 38 (4):373-5.

Rudy, A. C., P. M. Knight, D. C. Brater, and S. D. Hall. 1991. "Stereoselective metabolism of ibuprofen in humans: administration of R-, S- and racemic ibuprofen." *J Pharmacol Exp Ther* 259 (3):1133-9.

Tan, S. C., B. K. Patel, S. H. Jackson, C. G. Swift, and A. J. Hutt. 2003. "Influence of age on the enantiomeric disposition of ibuprofen in healthy volunteers." *Br J Clin Pharmacol* 55 (6):579-87.

U.S. Department of Health and Human Services. 2015. "VICH GL49(R): Guidance for Industry. Studies to evaluate the metabolism and residue kinetics of veterinary drugs in food-producing animals: validation of analytical methods used in residue depletion studies, revision at step 9 for implementation at step 7."
